# Medication adherence during the run‐in phase of clinical trials: A systematic review of methodological and reporting rigour

**DOI:** 10.1002/bcp.70178

**Published:** 2025-07-26

**Authors:** Non Davies, Bernard Vrijens, Daniel F. B. Wright, Dyfrig A. Hughes

**Affiliations:** ^1^ Centre for Health Economics and Medicines Evaluation, North Wales Medical School Bangor University Bangor UK; ^2^ AARDEX Group Liège Belgium; ^3^ Department of Public Health Liège University Liège Belgium; ^4^ Sydney Pharmacy School, Faculty of Medicine and Health University of Sydney Sydney Australia; ^5^ Department of Clinical Pharmacology & Toxicology St. Vincent's Hospital Sydney Australia

**Keywords:** enrichment, lead‐in period, lead‐in phase, medication adherence, run‐in period, run‐in phase, single‐blind placebo

## Abstract

**Aims:**

Trial run‐in phases are sometimes used to select adherent participants as a strategy to enrich trials, but the methods employed lack clear regulatory guidance and there are concerns about the transparency of reporting. This review aims to characterize the methods used for adherence measurement and public reporting of run‐in studies.

**Methods:**

The protocol is published on the Open Science Framework. Clinicaltrials.gov was searched using the terms “run*in”, “lead*in”, “enrichment” or “single‐blind placebo”. The Risk of Bias tool for Observational Adherence Studies (RoBOAS) and the ESPACOMP Medication Adherence Reporting Guideline (EMERGE) were used to assess methodological and reporting quality. A narrative synthesis of the evidence was undertaken.

**Results:**

In total, 249 studies were identified, 34 were included for the analysis, of which 8 specified adherence to be a main purpose of the run‐in. Most (20/34) used pill counts and of those using an explicit threshold for adherence, 13/31 used an 80% cut‐off. The reporting of the adherence measurement method, summary metric and method of data aggregation was complete in 23/34 run‐ins, but none publicly reported the adherence phase considered or presented any adherence data. All run‐ins were judged to be of poor methodological quality, and at critical risk of bias.

**Conclusions:**

The methodological and public reporting quality of medication adherence in run‐in phases of drug trials may compromise their intended purpose of improving the quality of the main trial. These findings have regulatory implications and support the need for guidelines on the measurement, analysis and reporting of adherence during the run‐in phase.

**What is already known about this subject**

- Trial run‐ins are used as an enrichment strategy in later‐phase clinical studies to select participants based on certain factors or characteristics, including their adherence to clinical trial investigational medicinal products.
- Run‐in phases have been critiqued for their public reporting deficiencies that preclude meaningful assessments of their impact on the validity of trial results.

**What this study adds**

- The methods used for measuring adherence in trial run‐ins are liable to bias, and the reporting lacks transparency, with clinical trial investigators consistently failing to publicly document any adherence data.
- The findings of this review should inform the design, conduct and reporting of future trial run‐ins that consider adherence.

## 1 INTRODUCTION

Trial run‐in phases are used as an enrichment strategy in later‐phase clinical studies to select participants based on certain factors or characteristics. This is done with the aim of reducing variability and increasing effect size.^1^, ^2^ Participants may typically be ineligible for randomization in the main trial based on a lack of adherence, placebo response or intolerance to the active medicine.^3^ Around 5% of randomized controlled trials include a run‐in phase,^4^ the benefits of which include lower rates of participant dropout from the main trial and increased statistical power.^5^ However, these come at the expense of reduced external validity, particularly if the individuals excluded following the run‐in are systematically different from those treated in routine clinical practice.^6^

The impact of medication nonadherence on trial outcomes may be mitigated by defining nonadherent patients as ineligible following a run‐in phase.^7^ Medication adherence is considered in 3 phases: initiation, implementation and persistence,^8^ but is often poorly measured, characterized and reported in clinical trials.^7^, ^9^ Undetected nonadherence may increase the dose requirement, compromise estimates of efficacy and the risk of harm^10^, ^11^ and necessitate an increase in the number of trial participants, ultimately contributing to further costs.^10^

There is currently no dedicated guidance on the run‐in phase from the Food and Drug Administration (FDA), European Medicines Agency or Medicines and Healthcare Products Regulatory Agency. The Consolidated Standards of Reporting Trials (CONSORT) statement also fails to mention the run‐in phase.^12^ Despite the FDA's guidance for Enrichment Strategies for Clinical Trials^2^ and the World Health Organization's guidance for Best Practices for Clinical Trials,^13^ both describing run‐in phases as an enrichment strategy to select adherent participants for the main trial, there is a lack of guidance about how best to measure, characterize and report adherence in the run‐in phase.

Reviews of medication adherence assessment and reporting in later‐phased trials have found significant heterogeneity in the definitions applied and the methods adopted.^7^, ^14^, ^15^ A recurring issue has been the underuse of least‐biased measures of adherence and application of suitable metrics. Previous reviews of trial run‐ins reported their frequency of use and transparency of reporting^4^, ^16^ but to our knowledge, there are no reports of the methodological and public reporting rigour of medication adherence in the run‐in phase of drug trials. This review therefore aims to: (i) identify phase 2 or 3 studies that use run‐ins to screen adherent participants for eligibility for the main trial; (ii) critically analyse the methods used for adherence measurement; and (iii) characterize the public reporting of medication adherence during the run‐in phase of these studies.

## 2 METHODS

### 2.1 Registration and protocol

The protocol for this review is registered on The Open Science Framework (https://doi.org/10.17605/OSF.IO/G8X42).

### 2.2 Eligibility criteria

#### 2.2.1 Inclusion criteria

We defined a trial run‐in phase as a distinct, pre‐enrolment period to the main trial, in which the run‐in population received the same treatment (active/placebo). Studies were included if participants' inclusion to the main trial was dependent on medication adherence during the run‐in phase.

#### 2.2.2 Exclusion criteria

Studies that described measuring medication adherence during the run‐in phase, but participants' inclusion in the main trial was not dependent on adherence during the run‐in phase were excluded. Studies that did not include full copies of the protocol and statistical analysis plan were also excluded.

### 2.3 Information sources

Studies were identified via the National Library of Medicine's registry of clinical trials ClinicalTrials.gov on 4 March 2024. ClinicalTrials.gov was chosen, as entries specify explicitly whether a trial includes a run‐in phase. Where available, publications relating to the included studies were identified by cross‐referencing the ClinicalTrials.gov unique identifier number with PubMed.

### 2.4 Search strategy

Studies were identified using the search terms “run*in”, “lead*in”, “enrichment”, “single‐blind placebo”, separated by the OR Boolean operator. Filters were applied to restrict the results to completed, phase 2 or 3 interventional studies, given that run‐ins are mainly used to enhance the likelihood of identifying a true treatment effect (efficacy).^4^ This may be less relevant for phase 4 studies (where the emphasis is typically more on clinical effectiveness and/or safety) or phase 1 studies, and so these were not included in the review. The results were also restricted to studies with published results, containing the protocol and statistical analysis plan. The study start date (first patient enrolled) was restricted to the period since 1 January 2010, chosen to coincide with improved reporting in ClinicalTrials.gov following the 2007 FDA Amendments Act.^17^

### 2.5 Selection process

The screening and selection of studies against the eligibility criteria were performed by 1 reviewer (N.D.). This was done initially using information from each study's entry in ClinicalTrials.gov, then using information from the study's protocol and statistical analysis plan. In the case of uncertainties, a second reviewer (D.H.) was consulted.

### 2.6 Data collection process

Data extraction was performed by 1 reviewer (N.D.), and in the case of uncertainties, a second reviewer (D.H.) was consulted. If studies referred to the *treatment period*, it was assumed that this included the run‐in phase—for instance, if the protocol described using pill counts to measure adherence during *the treatment period*, it was assumed that this was also true for the run‐in phase. Similarly, if studies did not state explicitly whether the adherence measurement method, summary metric and method of data aggregation was specific to the run‐in phase, this was inferred from the data and description given. In some instances, the measurement method (the method used to collect adherence data) was inferred from the summary metric (the metric used to quantify adherence data) (e.g., pill count was assumed if the summary metric referred to the number of tablets).

### 2.7 Data items

The following data were extracted: study year, Clinicaltrials.gov ID number, clinical trial phase, country, population, allocated treatments and dose, dosage frequency, run‐in type, main purpose of run‐in, run‐in duration, blinding during run‐in, number of patients included in run‐in, ineligible for main trial, enrolled in main trial, medication adherence phase, measure, metric, method of data aggregation and what was said about testing for adherence during the run‐in (including the use of any medication adherence thresholds for eligibility).

### 2.8 Methodological quality (run‐in phase risk of bias assessment)

The risk of bias tool for observational adherence studies (RoBOAS) was used to assess the methodological quality of the included run‐ins, given they are all, in effect, prospective cohort studies.^18^, ^19^ This tool incorporates the major points from the ESPACOMP Medication Adherence Reporting Guideline (EMERGE) guideline^20^ and the Timelines–Events–Objective–Sources (TEOS) Framework,^21^ as well as other potential sources of biases encountered in observational studies.^18^ The tool produces an overall bias judgement ranging from *low* to *critical* based on items across 4 domains considering bias related to: study design and implementation of study procedures, confounding factors, adherence outcome measurement method and public reporting, and data analysis and interpretation. Within each domain, items are rated across a scale of *fully present* to *fully absent*.

### 2.9 Quality of the public reporting of run‐ins

The EMERGE guidelines were used to assess the quality of public reporting of the included run‐ins.^20^ Specifically, the review assessed compliance with the 4 minimum reporting criteria; phases of medication adherence (as defined by the ABC taxonomy of adherence^8^), operational definitions (as defined by the TEOS Framework^21^), measures and results.

### 2.10 Synthesis methods

A narrative synthesis of the data was undertaken, with studies grouped for synthesis by frequencies—such as of the characteristics of the run‐in phase and the methods used. Information was tabulated and organized by study start year. Should the data allow, a meta‐analysis would be performed.

### 2.11 Reporting

Our reporting aimed to align with the principles of the Preferred Reporting Items for Systematic reviews and Meta‐Analyses (PRISMA)^22^ and the Synthesis Without Meta‐analysis (SWiM) guidelines.^23^

## 3 RESULTS

**FIGURE 1:**
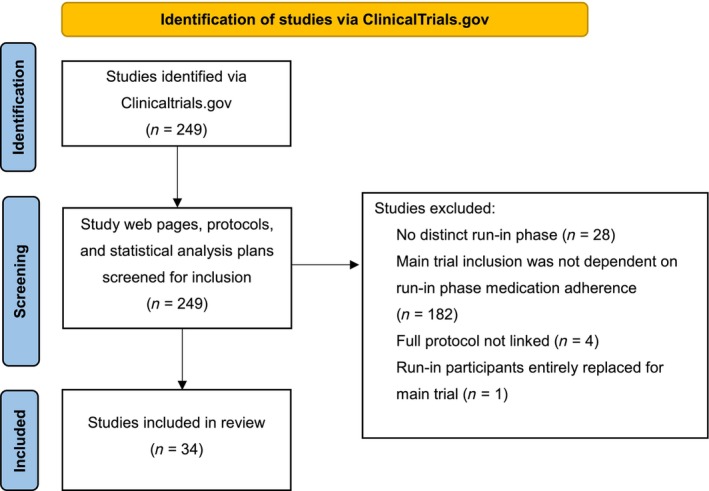
PRISMA flow diagram. Adapted from Page *et al*.^22^

A total of 249 studies were identified. After screening entries in ClinicalTrials.gov, protocols and statistical analysis plans, 215 studies were excluded for the reasons detailed in Figure 1. A total of 34 studies were included in the final review.^24^, ^25^, ^26^, ^27^, ^28^, ^29^, ^30^, ^31^, ^32^, ^33^, ^34^, ^35^, ^36^, ^37^, ^38^, ^39^, ^40^, ^41^, ^42^, ^43^, ^44^, ^45^, ^46^, ^47^, ^48^, ^49^, ^50^, ^51^, ^52^, ^53^, ^54^, ^55^, ^56^, ^57^ A meta‐analysis was not performed due to the heterogeneity across studies.

### 3.1 Study contexts

The details of each clinical trial are summarized in Table S1. Overall, 21 studies were phase 3,^25^, ^27^, ^28^, ^29^, ^31^, ^32^, ^34^, ^36^, ^37^, ^39^, ^40^, ^41^, ^42^, ^43^, ^44^, ^45^, ^46^, ^47^, ^48^, ^50^, ^55^ 12 were phase 2,^24^, ^26^, ^30^, ^33^, ^35^, ^38^, ^49^, ^51^, ^52^, ^53^, ^56^, ^57^ and 1 study was phase 1/2.^54^ These evaluated 26 medicines or combinations of medicines across 17 different diseases, with type 2 diabetes being the most common indication. The majority of studies (33/34) were sponsored by the (bio)pharmaceutical industry.

### 3.2 Run‐in phase characteristics

The characteristics of the run‐in phases are presented in Table S2. Of the 34 run‐ins included in this review, 8 specified medication adherence to be a main purpose of the run‐in,^27^, ^28^, ^31^, ^41^, ^50^, ^55^, ^56^, ^57^ whilst 11 specified other main purposes for the run‐in,^24^, ^25^, ^26^, ^29^, ^36^, ^37^, ^38^, ^40^, ^42^, ^43^, ^51^ and 15 did not specify a main purpose.^30^, ^32^, ^33^, ^34^, ^35^, ^39^, ^44^, ^45^, ^46^, ^47^, ^48^, ^49^, ^52^, ^53^, ^54^ Of those stating other main purposes, 8 aimed to ensure clinical or biochemical stability,^24^, ^27^, ^29^, ^38^, ^40^, ^42^, ^43^, ^56^ 3 served the purpose of wash‐out or elimination of the effects of current therapy or placebo,^25^, ^26^, ^31^ 3 established baseline values or incidence,^25^, ^27^, ^51^ 2 familiarized study participants with procedural or study techniques,^37^, ^50^ 3 excluded placebo responders or minimized response to placebo,^36^, ^40^, ^43^ and 1 monitored for safety.^57^

Nineteen studies used a placebo run‐in,^25^, ^26^, ^27^, ^28^, ^30^, ^31^, ^32^, ^34^, ^36^, ^39^, ^40^, ^41^, ^42^, ^43^, ^44^, ^45^, ^50^, ^55^, ^56^ 12 used an active run‐in,^24^, ^29^, ^37^, ^38^, ^46^, ^47^, ^48^, ^49^, ^51^, ^52^, ^53^, ^54^ and 3 used both active medication and placebo, either together^33^ or sequentially.^35^, ^57^ Fourteen run‐ins were single‐blind,^26^, ^28^, ^30^, ^31^, ^32^, ^34^, ^35^, ^36^, ^40^, ^41^, ^42^, ^43^, ^44^, ^45^ 9 were open‐label,^25^, ^27^, ^29^, ^37^, ^45^, ^46^, ^50^, ^56^, ^57^ 1 used a combination of single‐blind and open‐label sequentially,^33^ and 10 did not report blinding.^38^, ^39^, ^47^, ^48^, ^49^, ^51^, ^52^, ^53^, ^54^, ^55^ The median duration of the run‐in phases was 2 weeks (range, 12 days to 24 weeks).

Among the 15 studies that reported the participant exclusions from the main trial made as a result of the run‐in,^25^, ^26^, ^27^, ^28^, ^29^, ^30^, ^31^, ^36^, ^45^, ^49^, ^51^, ^52^, ^55^, ^56^, ^57^ 29.3% (2449/8333) of participants included in the run‐in were ineligible for the main trial. No data were available to show how many participants were ineligible due to nonadherence. A CONSORT diagram was available for 12 of the 15 studies for which a publication was identified and, of these, 7 referred to the run‐in phase.^26^, ^28^, ^29^, ^36^, ^49^, ^51^, ^55^ Only 6 studies referred to the run‐in phase within the tabulated data of participant flow in their ClinicalTrials.gov entries.^29^, ^30^, ^31^, ^49^, ^56^, ^57^

### 3.3 Medication adherence in the run‐in phase

The most common adherence measurement method used in the run‐ins was pill counts (20/34)^26^, ^27^, ^28^, ^31^, ^32^, ^33^, ^34^, ^35^, ^38^, ^39^, ^40^, ^41^, ^42^, ^43^, ^44^, ^45^, ^50^, ^51^, ^52^, ^57^ with the summary metric usually based on the number of doses taken expressed as a proportion of the expected number of doses taken (Table 1). The public reporting of the adherence measurement method, summary metric and method of data aggregation was complete in 23/34 run‐ins,^24^, ^25^, ^26^, ^27^, ^28^, ^31^, ^32^, ^33^, ^34^, ^35^, ^37^, ^39^, ^40^, ^41^, ^42^, ^43^, ^44^, ^45^, ^46^, ^50^, ^51^, ^56^, ^57^ with no run‐ins publicly reporting the medication adherence phase studied (as defined by the ABC taxonomy).^8^ For instance, in the largest of these run‐ins,^25^ adherence to placebo was measured in 1714 participants by entries in electronic diaries, quantified as the recorded number of doses taken, and summarized descriptively and by frequency.

**TABLE 1:** Medication adherence in the run‐in phase (EMERGE minimum reporting criteria 1a–1d).

Study reference	Main stated purpose of run‐in	Measure of adherence	Metric of adherence	Method of data aggregation	What was said about testing for adherence during run‐in
^24^	To achieve steady state on bronchodilation	eDiary	Percentage adherence based on vial count divided by the number of vials expected to be taken. The percent of *yes* responses to drug intake questions in eDiary also recorded.	Adherence summarized by treatment group.	Eligibility for randomization included adherence with the study drug during the run‐in phase (>75%) based upon eDiary entries.
^25^	To wash out any pre‐study corticosteroids or long‐acting bronchodilators; to establish forced expiratory volume in 1‐s baseline values	eDiary entries	Recording of doses taken.	Adherence analysed using descriptive statistics by study period and treatment group. The proportion of adherent *vs*. nonadherent subjects tabulated for each study period and treatment group.	Eligibility for randomization included adherence between 75 and 125% to placebo during the run‐in phase based upon eDiary entries.
^26^	To eliminate the effects of any previous antihypertensive therapy	Pill count	Percentage adherence based on number of capsules dispensed and counts of returned capsules at subsequent study visits.	Adherence categorized as: <80, 80–120 and >120%.	Adherence ≥80% during the run‐in phase based upon pill count is described as a study inclusion criterion.
^27^	To screen out nonadherent patients; to capture a *true* baseline incidence rate of hypoglycaemia; baseline continuous glucose monitoring profiles; optimal insulin use	Pill count	Percentage adherence based on number of tablets taken divided by number of tablets which should have been taken in same period.	Only descriptive statistics planned. Frequency distribution of patients with overall adherence between 80 and 120% (inclusive) reported, as well as patients outside this range. Overall adherence calculated as a weighted average of reported adherence. The sum of all reported adherence over the planned visits (disregarding of run‐in) divided by the total duration (until last visit where medication is returned).	Adherence between 80 and 120% to placebo during the run‐in phase is described as a study inclusion criterion.
^28^	To evaluate study drug adherence	Pill count	Percentage adherence based on tablet count divided by the expected number of tablets to be taken.	Adherence summarized by descriptive statistics and by the number of subjects in each category: <70, 70–130, >130%.	Adherence <70 or >130% to placebo drug during the run‐in phase is described as a study exclusion criterion.
^29^	To identify patients whose disease remained stable and in remission	Not reported	Not reported.	Not reported.	Nonadherence included as a reason for removal from run‐in.
^30^	Not reported	Vial/syringe count	Not reported.	Not reported.	Adherence during run‐in period assessed before randomization.
^31^	To wash out the placebo effect of the study drug and the influence of any prior antihypertensive drug treatment; to assess adherence in order to exclude subjects who are not sufficiently adherent	Pill count	Percentage adherence based on the number of doses of study drug for the treatment period divided by the duration of exposure to study drug for the treatment period.	Percentage adherence categorized as: ≤69.9, 70.0 to ≤79.9, 80.0 to ≤89.9, ≥90.0%	Adherence <70 or >130% with the study drug during the run‐in phase is described as a study exclusion criterion.
^32^	Not reported	Pill count	Percentage of days adherent based on the number of days that the patient was adherent divided by the total number of days that the patient was planned to take the medicine.	Adherence data summarized descriptively as a quantitative variable (number, mean, SD, median, minimum and maximum). Percentage of patients whose adherence is <80% summarized.	Eligibility for randomization included adherence with the study drug during the run‐in phase (>80%) based upon tablet count, and also “based on the opinion of the investigator”.
^33^	Not reported	Pill count	Percentage of days adherent based on the number of days that the patient was on therapy divided by the total number of days that the patient should be on therapy.	Summary statistics provided on percent adherent by treatment group.	Adherence 80% with trial medication between visits 3 and 4 is described as a study inclusion criterion.
^34^	Not reported	Pill count	Percentage of days adherent based on the number of days that the patient was adherent divided by the total number of days that the patient was planned to take the medicine.	Adherence data summarized descriptively as a quantitative variable (number, mean, SD, median, minimum and maximum). Percentage of patients whose adherence is <80% summarized.	Eligibility for randomization included adherence with the study drug during the run‐in phase (>80%) based upon tablet count, and also “in the opinion of the investigator”.
^35^	Not reported	Pill count	Percentage adherence based on number of capsules taken divided by expected number of capsules to be taken.	Adherence summarized by randomized treatment group and total. No inferential statistics presented. Adherence summarized for both the run‐in and double‐blind periods separately. Adherence during the double‐blind period categorized into: <75, ≥75, ≤133 and >133%. Adherence during the run‐in period categorized into: <85 and ≥85%.	Adherence ≥85% with the study drug during the run‐in phase is described as a study inclusion criterion.
^36^	To exclude placebo responders	Patient diary	Percentage of days adherent during run‐in period based on the number of days with “Adherence of study drug” in diary of “Yes” during run‐in period divided by the duration of exposure to study drug in run‐in period.	Not reported.	Adherence ≥75% with the study drug during the run‐in phase is described as a study inclusion criterion.
^37^	To ascertain that the subject acceptably performs all necessary procedures and can be eligible for randomization	Dose counter	Percentage adherence based on the recorded number of puffs of medication divided by the expected number of puffs.	Summary statistics provided on percent adherence by treatment group	Adherence <80% with the study drug during the run‐in phase based upon dose counter readings is described as a study exclusion criterion.
^38^	To allow suppression of endogenous testosterone production; to allow the oral testosterone undecanoate to reach steady state	Pill count	Adherence based on actual number of doses taken divided by the expected number of doses taken.	Not reported.	Adherence >80% with the study drug during run‐in phase is required to proceed to the main study.
^39^	Not reported	Pill count	Percentage adherence based on number of tablets taken divided by the number of tablets.	Only descriptive statistics provided. Frequency distribution of patients with adherence between 80 and 120% presented by visit.	Adherence to placebo during the run‐in period should be between 80 and 120%, and patients should be carefully interviewed and, if necessary, re‐informed about the purpose and the conduct of the trial if outside of this range. Unreliable patients should not be randomized “at the discretion of the investigator”.
^40^	To stabilize haemoglobin A1c; to minimize response to placebo and effects inherent to participation in a controlled clinical trial	Pill count	Percentage of days adherent based on the number of days that the patient was adherent divided by the total number of days that the patient was planned to take the medicine.	Adherence data summarized descriptively as a quantitative variable (number, mean, SD, median, minimum and maximum). Percentage of patients whose adherence is <80% summarized.	Eligibility for randomization included adherence to placebo during the run‐in phase (≥80%) based upon tablet count, and also “at the investigator's discretion”.
^41^	To assess patients' adherence to the study medication	Pill count	Percentage of days adherent based on the number of days that the patient was adherent divided by the total number of days that the patient was planned to take the medicine.	Adherence data summarized descriptively as a quantitative variable (number, mean, SD, median, minimum and maximum). Percentage of patients whose adherence is <80% summarized.	Eligibility for randomization included adherence with the study drug during the run‐in phase (>80%) based upon tablet count, and also “based on the opinion of at the investigator”.
^42^	Lantus titration	Pill count	Percentage of days adherent based on the number of days that the patient was adherent divided by the total number of days that the patient was planned to take the medicine.	Adherence data summarized descriptively as a quantitative variable (number, mean, SD, median, minimum and maximum). Percentage of patients whose adherence is <80% summarized.	Eligibility for randomization included adherence with the study drug during the run‐in phase (>80%) based upon tablet count, and also “based on the opinion of at the investigator
^43^	To stabilize haemoglobin A1c; to minimize response to placebo and effects inherent to participation in a controlled clinical trial	Pill count	Percentage of days adherent based on the number of days that the patient was adherent divided by the total number of days that the patient was planned to take the medicine.	Adherence data summarized descriptively as a quantitative variable (number, mean, SD, median, minimum and maximum). Percentage of patients whose adherence is <80% summarized.	Eligibility for randomization included adherence to placebo during the run‐in phase (≥80%) based upon tablet count, and also “at the investigator's discretion”.
^44^	Not reported	Pill count	Percentage of days adherent based on the number of days that the patient was adherent divided by the total number of days that the patient was planned to take the medicine.	Adherence data summarized descriptively as a quantitative variable (number, mean, SD, median, minimum and maximum). Percentage of patients whose adherence is <80% summarized.	Adherence <80% with the study drug during the run‐in phase based upon tablet count is described as a study exclusion criterion.
^45^	Not reported	Pill count	Percentage of days adherent based on the number of days that the patient was adherent divided by the total number of days that the patient was planned to take the medicine.	Adherence data summarized descriptively as a quantitative variable (number, mean, SD, median, minimum and maximum). Percentage of patients whose adherence is <80% summarized.	Eligibility for randomization included adherence with the study drug during the run‐in phase (>80%) based upon tablet count, and also “in the opinion of the investigator”.
^46^	Not reported	Dose counter	Percentage adherence based on total number of inhalations taken divided by expected inhalations multiplied by duration of exposure.	Percentage of adherence summarized categorically and with descriptive statistics.	Eligibility for randomization included adherence with the study drug during the run‐in phase, defined as use of run‐in medication on at least 4 of the last 7 consecutive days of the run‐in period.
^47^	Not reported	eDiary	Not reported.	Not reported.	Adherence >70% with the study drug during the run‐in period is described as a study inclusion criterion.
^48^	Not reported	eDiary	Not reported.	Not reported.	Adherence >70% with the study drug during the run‐in phase is described as a study inclusion criterion.
^49^	Not reported	Not reported	Not reported.	Not reported.	Nonadherence included as a reason for removal from run‐in.
^50^	To familiarize the patient with the procedures for study medication intake prior to randomization, giving an opportunity for assessing the patient ability to be adherent with the study medication intake	Pill count	Percentage adherence based on the number of tablets taken divided by the number of tablets which should have been taken.	Descriptive statistics provided. Descriptive summary of the percentage of adherence and frequency distribution of patients in each category: <75, 75–125, >125%, incalculable.	Adherence between 75 and 125% to placebo during the run‐in phase is described as a study inclusion criterion.
^51^	To allow for collection of baseline eDiary data	Pill count	Percentage adherence based on the number of tablets taken divided by the number of tablets expected to be taken.	Adherence summarized using descriptive statistics and categorized as: <80, ≥80–100, >100–<120, ≥120%. Adherence also presented in a by‐subject data listing.	Eligibility for randomization included adherence ≥75% to standard of care during the run‐in phase.
^52^	Not reported	Pill count	Not reported.	Not reported.	Adherence <90% with the study drug during the run‐in phase is described as a study exclusion criterion.
^53^	Not reported	Not reported	Not reported.	Not reported.	Adherence 80% during the run‐in phase must be verified prior to randomization.
^54^	Not reported	Pill diary	Not reported.	Not reported.	Eligibility for randomization included participants receiving at least 75% of doses during run‐in phase.
^55^	To assess their ability to adhere to daily eye drops	Adherence calendar and count of unused ampules	Adherence based on days adherent divided by total days since receiving study medication.	Not reported.	Eligibility for randomization included adherence to artificial tears drug during the run‐in phase (>90%) based upon review of adherence calendar and unused ampule count.
^56^	To confirm a stable asthma medication regimen; to assess adherence to the dosing schedule, after which eligible subjects will be randomized	Smart bottle and visual verification of unused pill count	Percentage adherence based on number of tablets returned divided by the number of tablets expected to be taken.	Descriptive statistics of the percentage adherence provided by treatment. Adherence categorized in a frequency table as: <60, ≥60, <80, ≥80, <120, ≥90 and <105%.	Adherence >85% to placebo during the run‐in phase based upon smart bottle is described as a study inclusion criterion.
^57^	To monitor for safety and study drug adherence	Pill count	Percentage adherence based on total amount of study drug administered divided by study drug expected to be administered.	Descriptive statistics and the number and percentage of subjects belonging to the adherence categories: <75, ≥75–<90, ≥90%, provided by treatment group.	Prior to randomization, subjects must have at least 7 consecutive daily doses of study drug.

Abbreviations: eDiary, electronic diary; SD, standard deviation.

All but 3 studies^29^, ^30^, ^49^ described using a medication adherence eligibility threshold as a criterion for proceeding to the trial. Of those using a threshold, most (13 run‐ins) used 80%.^26^, ^32^, ^33^, ^34^, ^37^, ^38^, ^40^, ^41^, ^42^, ^43^, ^44^, ^45^, ^53^ Eight studies reported using both a threshold and the “investigator's discretion or opinion” when deciding whether to allow patients to proceed with the main trial.^32^, ^34^, ^39^, ^40^, ^41^, ^42^, ^43^, ^45^ No studies reported medication adherence data for the run‐in phase, and only 4/34 studies reported adherence data for the main trial.^24^, ^37^, ^55^, ^56^

### 3.4 Quality of public reporting

Data corresponding with the EMERGE guideline minimum reporting criteria are presented in Table 1. Compliance with the EMERGE guideline was poor, with no run‐ins publicly reporting 2 out of the 4 minimum reporting criteria outlined by the guidance (phases of medication adherence and results).^20^ All but 3 run‐ins reported the adherence measurement method used,^29^, ^49^, ^53^ but none of these studies considered each adherence phase studied, nor the performance of the measures. Additionally, despite all but 8 run‐ins publicly reporting the operational definition of adherence^29^, ^30^, ^47^, ^48^, ^49^, ^52^, ^53^, ^55^ (as defined by the TEOS framework),^21^ none did this within the context of an adherence phase.

### 3.5 Methodological quality (run‐in risk of bias assessment)

All run‐ins included in this review were judged to be of poor methodological quality, with all studies achieving a *critical* risk of bias judgement across the individual RoBOAS domains, as well as a *critical* overall risk of bias judgement.

## 4 DISCUSSION

We identified 34 clinical trials reporting a run‐in period, of which 8 specified medication adherence to be a main purpose. However, run‐ins exhibited poor methodological quality regarding adherence, with many being too short in duration, using methods of measurement that are liable to bias, or not providing adequate publicly accessible information.

Our findings compare with a previous review that aimed to characterize the frequency, characteristics and reporting of randomized trials with run‐in periods.^4^ They reported a median of 16% of participants being excluded from the run‐in (compared with 28% in our review), with both reviews reporting a median run‐in duration of 14 days. Collister *et al*.^16^ reviewed run‐ins of randomized trials in chronic diseases and reported more studies excluding nonadherent participants (42.9%) than we found in our review (23.5%); although this may be accounted, to an extent, by differences between reviews in their inclusion criteria. It should also be noted that medication nonadherence is not the sole exclusion criterion defining ineligibility to continue with the main trial.

Deficiencies in the reporting of run‐ins have also been noted previously^4^, ^16^ with 52% of identified studies reporting the number of participants excluded from the run‐in^4^; only slightly higher than our rate of 44%. Collister *et al*.,^16^ in their review of 42 trials, reported that 67% of studies referred to the run‐in phase in the CONSORT diagram (58% in our review), with a weakness of the CONSORT guidance being that it does not refer to the run‐in phase.^12^ The issue of incomplete reporting of CONSORT diagrams has been highlighted previously,^58^ with Laursen *et al*.^4^ highlighting that reporting the exclusions made during the run‐in is essential for the consideration of its impact on the external validity of the trial. Finally, Laursen *et al*.^4^ noted that only 8% of run‐ins had complete reporting, which is significantly lower than the 32% found in our review, although both studies judged reporting standards in different ways.

In the context of medication adherence in clinical trials more generally—extending beyond run‐ins—a review of drug registration trials considered by the European Medicines Agency, found over 99% of 253 studies to have measured adherence, with 90.2% using pill or dose counts.^7^ This is despite well‐recognized biases inherent to pill counts, especially arising from drug intake being implied from return rates, and the lack of granularity in understanding adherence patterns over time.^18^, ^59^, ^60^ In contrast to Mantila *et al*.,^7^ none of our included run‐ins reported any data on medication adherence. This inadequacy of reporting in the public domain may be linked to the inconsistent transfer of data to trial statisticians. Within main trials, at least, adherence data are not used routinely for central monitoring purposes, but rather, they are often used by trial investigators to classify participants as being adherent (or not).^9^ Despite considerable attention usually being placed upon medication adherence in study protocols and the monitoring of trial participants, this same attention is seemingly lost in the subsequent reporting of adherence.^14^

The ubiquitous use of a threshold, most often based on 80% of assumed doses taken, has been considered inappropriate due to a lack of validation. A single threshold cannot accommodate differences in clinical contexts, drug forgiveness or adherence patterns.^7^, ^9^, ^61^, ^62^ Despite this, adherence eligibility thresholds were used as a criterion for participants to proceed to the main trial in 88% of the run‐ins included in our review.

Although the investigators of the trials included in our review were cognisant of the importance of medication adherence (as evidenced by all studies measuring adherence during the run‐in), the quality of how run‐in adherence was measured and reported was poor, rendering them liable to methodological bias and deficient with respect to transparency of public reporting.

Our review has advantages in using ClinicalTrials.gov to identify relevant trials. Unlike electronic databases of citations such as PubMed and EMBASE, identifying run‐in trials in ClinicalTrials.gov is easily facilitated using a search filter and is more specific. Indeed, only 6 of the 19 trial publications were identified using these 2 databases when applying the same search terms. Nonetheless, our search may have limited sensitivity, and so miss some relevant studies. While ClinicalTrials.gov is a comprehensive registry of clinical trials worldwide, there have been concerns about compliance of sponsors and trial investigators with legal requirements of submitting details of their trials.^17^ Identified studies that did not include full copies of the protocol or statistical analysis plan were excluded from our review, and therefore the percentage of underreporting may be even higher than we found. The inception of our review (defined by the date of first patient enrolled) was such that analyses and publications would postdate the publication of the taxonomy for describing and defining adherence to medications.^8^ Studies including run‐ins that started enrolment prior to 2010 and that were excluded from this review might nonetheless be methodologically sound.

Run‐in duration was generally short (median of 2 weeks) which may increase the risk of misclassification error; that is, categorizing run‐in participants as nonadherent when in fact they are adherent, and vice versa. For instance, 80% adherence to once‐daily treatment over 2 weeks is associated with a standard error of 10.7%. Increasing the run‐in to 4 weeks reduces the standard error to 7.6%. As such, due regard to the duration of run‐ins is advised when considering the consequence of falsely including or excluding trial participants on the basis of their adherence.

More fundamentally, excluding participants based on run‐in phase adherence may compromise the external validity of the trials, limiting the generalizability of their results to wider clinical practice. Selecting the trial population based on an arbitrary adherence threshold might not be reflective of the true adherence rates observed in clinical practice.^6^, ^63^

The findings of this review should inform the design, conduct and reporting of future run‐ins. We found that despite medication adherence being measured frequently during the run‐in phase of drug trials, the methods used are often liable to bias, with public reporting incomplete. Our review highlights that methodological deficiencies in run‐in phases undermine their intended purpose of improving the quality of the main trial. Future research should focus on improving the methodological quality of run‐ins, with a particular focus on using unbiased measures of adherence and appropriate reporting standards. The development of guidelines for conducting run‐ins should facilitate this, as well as have regulatory implications for best practice.

## AUTHOR CONTRIBUTIONS

N.D., B.V., D.F.B.W. and D.A.H. made substantial contributions to conception, design and interpretation of data; N.D. collected and analysed the data and drafted the manuscript; B.V., D.F.B.W. and D.A.H. revised the manuscript critically for important intellectual content; N.D., B.V., D.F.B.W. and D.A.H. all gave their final approval of the version to be published; and agreed to be accountable for all aspects of the work in ensuring that questions related to the accuracy or integrity of any part of the work are appropriately investigated and resolved.

## CONFLICT OF INTEREST STATEMENT

The authors declare no conflicts of interest.

## Supporting information


**TABLE S1** Study context.
**TABLE S2** Run‐in phase characteristics.

## Data Availability

Data are publicly available via https://clinicaltrials.gov/.
