# Supplementary material for: Medication adherence during the run‐in phase of clinical trials: A systematic review of methodological and reporting rigour
Source: Br J Clin Pharmacol. 2025 Jul 26;91(12):3353–65. doi: 10.1002/bcp.70178 (PMC12648378; doi:10.1002/bcp.70178)
Supplement: Supplementary file 1 — TABLE S1 Study context. TABLE S2 Run‐in phase characteristics. [file BCP-91-3353-s001.docx]

Supplementary Appendix Table 1 – Study context

| Study reference | Study start year | Clinicaltrials.gov ID number | Study phase | Country | Population | Allocated treatments and dose | Dosage frequency |
| --- | --- | --- | --- | --- | --- | --- | --- |
| (24) | 2011 | [NCT03937479](https://clinicaltrials.gov/study/NCT03937479) | 2. | United States. | Patients with moderate to severe Chronic Obstructive Pulmonary Disease on a background of once daily tiotropium. | Ensifentrine (0.375mg) + tiotropium (dose not reported).  Ensifentrine (0.75mg) + tiotropium (dose not reported).  Ensifentrine (5mg) + tiotropium (dose not reported).  Ensifentrine (3mg) + tiotropium (dose not reported).  Placebo + tiotropium (dose not reported). | Twice daily. |
| (25) | 2013 | [NCT02495168](https://clinicaltrials.gov/study/NCT02495168) | 3. | United States. | Adolescent and adult participants with chronic but stable asthma. | Generic Budesonide/Formoterol Fumarate Dihydrate (2 X 80μg/4.5μg).  Symbicort (Budesonide/Formoterol Fumarate Dihydrate) (2 X 80μg/4.5μg).  Placebo. | Twice daily. |
| (26) | 2015 | [NCT02603809](https://clinicaltrials.gov/study/NCT02603809) | 2. | United States, Canada, Israel, and Puerto Rico. | Participants with grade 1 or 2 essential hypertension. | Aprocitentan (5mg).  Aprocitentan (10mg).  Aprocitentan (25mg).  Aprocitentan (50mg).  Lisinopril (20mg).  Placebo. | Once daily. |
| (27) | 2015 | [NCT02414958](https://clinicaltrials.gov/study/NCT02414958) | 3. | United States, Australia, Austria, Belgium, Canada, Czechia, Denmark, Finland, France, Germany, Netherlands, Norway, Poland, Spain, Sweden. Taiwan, United Kingdom. | Participants with Type 1 Diabetes Mellitus who are already using either an insulin regimen of multiple daily injections or continuous subcutaneous insulin infusion. | Empagliflozin (10mg).  Empagliflozin (25mg).  Placebo. | Once daily. |
| (28) | 2015 | [NCT02480764](https://clinicaltrials.gov/study/NCT02480764) | 3. | China. | Chinese subjects with essential hypertension. | Azilsartan medoxomil (40mg)  Azilsartan medoxomil (80mg).  Valsartan (160mg). | Once daily. |
| (29) | 2015 | [NCT02373813](https://clinicaltrials.gov/study/NCT02373813) | 3. | Canada, United States, Argentina, Bulgaria, Czech Republic, Spain, France, Germany, Greece, Hungary, Italy, Mexico, Poland, Portugal, and South Africa. | Participants with rheumatoid arthritis who were on etanercept plus methotrexate therapy. | Oral methotrexate (from 10 to 25 mg) + placebo + folic acid as standard of care.  Etanercept (50mg) + placebo + folic acid as standard of care.  Etanercept (50 mg) + methotrexate (from 10 to 25 mg) + folic acid as standard of care. | Once weekly. |
| (30) | 2015 | [NCT02311673](https://clinicaltrials.gov/study/NCT02311673) | 2. | United States. | Obese participants with Prader-Willi syndrome on tolerability, weight loss, and hyperphagia-related behavior. | Setmelanotide (0.5mg).  Setmelanotide (1.5mg).  Setmelanotide (2.5mg).  Placebo. | Once daily. |
| (31) | 2016 | [NCT02791438](https://clinicaltrials.gov/study/NCT02791438) | 3. | Japan. | Pediatric patients aged 6 to less than 16 years with hypertension. | Azilsartan (2.5-20mg) in participants < 50 kg.  Azilsartan (5-40mg) in participants ≥ 50 kg. | Once daily. |
| (32) | 2016 | [NCT02926937](https://clinicaltrials.gov/study/NCT02926937) | 3. | United States, Canada, Mexico. | Participants with Type 2 Diabetes Mellitus who have inadequate glycemic control on diet and exercise. | Sotagliflozin (400mg).  Sotagliflozin (200mg).  Placebo. | Once daily. |
| (33) | 2016 | [NCT02720081](https://clinicaltrials.gov/study/NCT02720081) | 2. | Not reported. | Adults with persistent asthma that is uncontrolled while receiving montelukast alone. | MK-1029 (150mg) + Montelukast (10mg).  Placebo + Montelukast (10mg). | Once daily. |
| (34) | 2016 | [NCT02926950](https://clinicaltrials.gov/study/NCT02926950) | 3. | United States, Canada, Hungary, Slovakia. | Participants with Type 2 Diabetes Mellitus who have inadequate glycemic control with metformin. | Sotagliflozin (400mg) and Metformin.  Placebo and Metformin. | Once daily. |
| (35) | 2016 | [NCT02743949](https://clinicaltrials.gov/study/NCT02743949) | 2. | Belgium, Bulgaria, Czechia, Estonia, Poland, United Kingdom. | Subjects with symptomatic gastro-esophageal reflux disease who have a partial response following treatment with a high dose of proton pump inhibitor | Vonoprazan (20mg).  Vonoprazan (40mg).  Esomeprazole (40mg). | Once daily. |
| (36) | 2016 | [NCT02954848](https://clinicaltrials.gov/study/NCT02954848) | 3. | Japan. | Participants with non-erosive gastroesophageal reflux disease. | TAK-438 (10mg).  Placebo. | Once daily. |
| (37) | 2016 | [NCT02741271](https://clinicaltrials.gov/study/NCT02741271) | 3. | Not reported. | Children (5 to 11 years of age) with persistent asthma. | Furoate/formoterol fumarate (100/10mcg).  Mometasone furate (100 mcg). | Twice daily. |
| (38) | 2016 | [NCT02921386](https://clinicaltrials.gov/study/NCT02921386) | 2. | United States. | Adult hypogonadal male subjects. | Meal plan A (fasting) + Oral Testosterone Undecanoate (237mg).  Meal plan B (15gm fat + Oral Testosterone Undecanoate (237mg).  Meal plan C (30gm fat, reference meal) + Oral Testosterone Undecanoate (237mg).  Meal plan D (45gm fat) + Oral Testosterone Undecanoate (237mg).  Meal plan E (FDA high-calorie, high-fat) + Oral Testosterone Undecanoate (237mg). | Twice daily. |
| (39) | 2016 | [NCT02897349](https://clinicaltrials.gov/study/NCT02897349) | 3. | China. | Chinese patients with Type 2 Diabetes Mellitus with insufficient glycaemic control. | Linagliptin (5mg).  Placebo. | Once daily. |
| (40) | 2017 | [NCT03242252](https://clinicaltrials.gov/study/NCT03242252) | 3. | United States, Argentina, Brazil, Canada, Colombia, Germany, Hungary, Israel, Italy, Mexico, Poland, Romania, Russian Federation, South Africa, Spain, Ukraine. | Participants with Type 2 Diabetes Mellitus and moderate renal impairment who have inadequate glycemic control. | Sotagliflozin (200mg).  Sotagliflozin (400mg).  Placebo. | Once daily. |
| (41) | 2017 | [NCT03351478](https://clinicaltrials.gov/study/NCT03351478) | 3. | Bulgaria, Canada, Czechia, France, Italy, Latvia, Mexico, Russian Federation, Slovakia, Spain, United Kingdom, United States. | Participants with Type 2 Diabetes who have inadequate glycemic control on dipeptidyl peptidase 4 inhibitor with or without metformin | Sotagliflozin (400mg).  Empagliflozin (25mg).  Placebo. | Once daily. |
| (42) | 2017 | [NCT03285594](https://clinicaltrials.gov/study/NCT03285594) | 3. | United States, Bulgaria, Canada, Czech Republic, France, Hungary, Slovakia, United Kingdom. | Patients with Type 2 Diabetes Mellitus using any types of basal insulin alone or in combination with up to 2 oral antidiabetes drugs. | Sotagliflozin (200mg).  Sotagliflozin (400mg).  Placebo. | Once daily. |
| (43) | 2017 | [NCT03242018](https://clinicaltrials.gov/study/NCT03242018) | 3. | United States, Argentina, Brazil, Colombia, Germany, Hungary, Israel, Italy, Mexico, Poland, Romania, Russian Federation, South Africa, Spain, Ukraine. | Patients With Type 2 Diabetes Mellitus and Severe Renal Impairment Who Have Inadequate Glycemic Control. | Sotagliflozin (200mg).  Sotagliflozin (400mg).  Placebo. | Once daily. |
| (44) | 2017 | [NCT03066830](https://clinicaltrials.gov/study/NCT03066830) | 3. | United States, Bulgaria, Estonia, Hungary, Republic of Korea, Poland, Romania, Slovakia, Ukraine, United Kingdom. | Participants with Type 2 Diabetes who have inadequate glycemic control with a Sulfonylurea alone or in combination with Metformin. | Sotagliflozin (400mg).  Placebo. | Once daily. |
| (45) | 2017 | [NCT03332771](https://clinicaltrials.gov/study/NCT03332771) | 3. | United States, Bulgaria, Hungary, Slovakia. | Patients with Type 2 Diabetes Mellitus who have inadequate control with metformin. | Sotagliflozin (200 mg).  Sotagliflozin (400mg).  Glimepiride (titrated up to 6mg).  Placebo. | Once daily. |
| (46) | 2017 | [NCT03248128](https://clinicaltrials.gov/study/NCT03248128) | 3. | United States, Argentina, Bulgaria, Canada, China, Germany, Hungary, Italy, Japan, Lithuania, Mexico, Poland, Romania, Russian Federation, South Africa, Spain | Participants between 5 and 17 years of age with asthma currently uncontrolled on inhaled corticosteroids. | Fluticasone furoate/vilanterol (50/25 mcg for subjects aged 5-11 years of age and 100/25 for subjects aged 12-17 years of age).  Fluticasone furoate (50 mcg for subjects aged 5-11 years of age and 100mcg for subjects aged 12-17 years of age). | Once daily. |
| (47) | 2017 | [NCT03280550](https://clinicaltrials.gov/study/NCT03280550) | 3. | United States, Canada, Czechia, Germany, Mexico, Poland, Portugal, Russian Federation, Ukraine, United Kingdom. | Participants with chronic rhinosinusitis with nasal polyps who have had an inadequate response to standard-of-care treatments. | Omalizumab  (from 75 to 600mg).  Placebo. | Once every 2 or 4 weeks. |
| (48) | 2017 | [NCT03280537](https://clinicaltrials.gov/study/NCT03280537) | 3. | United States, Belgium, Finland, France, Hungary, Mexico, Poland, Russian federation, Spain, Ukraine. | Adult patients with chronic rhinosinusitis with nasal polyps who have had an inadequate response to standard-of-care treatments. | Omalizumab (from 75 to 600mg).  Placebo. | Once every 2 or 4 weeks. |
| (49) | 2017 | [NCT03023878](https://clinicaltrials.gov/study/NCT03023878) | 2. | United States, Canada, France, Germany, Spain, United Kingdom. | Adults with newly diagnosed high-risk diffuse large B-cell lymphoma. | Blinatumomab (9 µg/day for 7 days, 28 µg/day for 7 days, 112 µg/day for 6 weeks).  Optional 4-week Cycle 2 of blinatumomab available for participants whose disease did not progress (9 µg/day for 7 days, 28 µg/day for 7 days, 112 µg/day for 14 days). | Once daily. |
| (50) | 2018 | [NCT03429543](https://clinicaltrials.gov/study/NCT03429543) | 3. | United States, Argentina, Brazil, Canada, China, Colombia, Germany, Israel, Republic of Korea, Mexico, Puerto Rico, Russian Federation, Thailand, United Kingdom. | Children and adolescents (10 to 17 years) with Type 2 Diabetes Mellitus currently taking metformin, insulin or both drugs. | Empagliflozin (10mg).  Linagliptin (5mg).  Placebo.  Re-randomisation at weeks 14 and 26. | Once daily. |
| (51) | 2018 | [NCT03683576](https://clinicaltrials.gov/study/NCT03683576) | 2. | United States, Austria, Belgium, Canada, Czechia, France, Germany, Poland, Spain, Ukraine, United Kingdom. | Subjects with moderate to severe asthma and an eosinophilic phenotype. | GB001 (20mg).  GB001 (40mg).  GB001 (60mg).  Placebo. | Once daily. |
| (52) | 2018 | [NCT03611010](https://clinicaltrials.gov/study/NCT03611010) | 2. | United States. | Hypercholesterolemic patients previously controlled on oral atorvastatin. | Atorvastatin (from 2 to 5mg) for subjects taking 10mg of atorvastatin during the lead-in.  Atorvastatin (from 4 to 10mg) for subjects taking 20mg of atorvastatin during the lead-in.  Atorvastatin (from 8 to 20mg) for subjects taking 40mg of atorvastatin during the lead-in.  Standard care. | Once daily. |
| (53) | 2018 | [NCT03614923](https://clinicaltrials.gov/study/NCT03614923) | 2. | United States. | Adults with chronic rhinosinusitis with nasal polyps. | Etokimab (300mg loading dose, then 150mg every 4 weeks) + mometasone furoate nasal spray of two actuations (50 μg/actuation).  Etokimab (300mg loading dose then 150mg every 8 weeks) + mometasone furoate nasal spray of two actuations (50 μg/actuation).  Placebo + mometasone furoate nasal spray of two actuations (50 μg/actuation). | Every 4 or 8 weeks. |
| (54) | 2018 | [NCT03792750](https://clinicaltrials.gov/study/NCT03792750) | 1/2. | China. | Chinese Patients with advanced malignant solid tumors. | BMS-986205 (100mg) + Nivolumab (480mg). | Once daily (BMS-986205) and once every 4 weeks (Nivolumab). |
| (55) | 2018 | [NCT03334253](https://clinicaltrials.gov/study/NCT03334253) | 3. | United States. | Children aged 5 to less than 13 years with myopia. | Atropine (0.01%). | One eye drop once daily. |
| (56) | 2019 | [NCT04046939](https://clinicaltrials.gov/study/NCT04046939) | 2. | United States. | Subjects with eosinophilic asthma. | Dexpramipexole (37.5mg).  Dexpramipexole (75mg).  Dexpramipexole (150mg).  Placebo. | Twice daily. |
| (57) | 2019 | [NCT04026165](https://clinicaltrials.gov/study/NCT04026165) | 2. | United States, Japan, Canada, New Zealand, and Australia. | Subjects with Type 2 Diabetes Mellitus and moderate to advanced diabetic kidney disease. | Selonsertib (18mg).  Placebo. | Once daily. |

Supplementary Appendix Table 2 – Run-in phase characteristics

| Study reference | Clinicaltrials.gov ID number | Reference(s) for main publication(s) | Type | Duration | Blinding | Number of patients:  Included in run-in;  Ineligible for main trial; Proceeded to main trial |
| --- | --- | --- | --- | --- | --- | --- |
| (24) | [NCT03937479](https://clinicaltrials.gov/study/NCT03937479) | (58) | Active | 2 weeks | Open-label | Not specified  Not specified  416 randomised |
| (25) | [NCT02495168](https://clinicaltrials.gov/study/NCT02495168) | None identified | Placebo | 2 weeks | Open-label | 1,714 enrolled  567 excluded  1,147 randomised |
| (26) | [NCT02603809](https://clinicaltrials.gov/study/NCT02603809) | (59) | Placebo | 4-6 weeks | Single-blind | 996 enrolled  506 excluded  490 randomised |
| (27) | [NCT02414958](https://clinicaltrials.gov/study/NCT02414958) | (60,61)‌ | Placebo | 2 weeks | Open-label | 1,015 enrolled  285 excluded  730 randomised |
| (28) | [NCT02480764](https://clinicaltrials.gov/study/NCT02480764) | (62) | Placebo | 2 weeks | Single-blind | 949 enrolled  337 excluded  612 randomised |
| (29) | [NCT02373813](https://clinicaltrials.gov/study/NCT02373813) | (63–66)‌ | Active | 24 weeks | Open-label | 371 enrolled  118 excluded  253 randomised |
| (30) | [NCT02311673](https://clinicaltrials.gov/study/NCT02311673) | None identified | Placebo | 2 weeks | Singe-blind. | 40 enrolled  0 excluded  40 randomised |
| (31) | [NCT02791438](https://clinicaltrials.gov/study/NCT02791438) | None identified | Placebo | 2 weeks. | Single-blind | 27 enrolled  0 excluded  27 enrolled into main trial (not randomised) |
| (32) | [NCT02926937](https://clinicaltrials.gov/study/NCT02926937) | None identified | Placebo | 2 weeks | Single- blind | Not specified  Not specified  399 randomised |
| (33) | [NCT02720081](https://clinicaltrials.gov/study/NCT02720081) | None identified | Active and placebo (both throughout) | 2 weeks | Single-blind (placebo) and open-label (active) | Not specified  Not specified  142 randomised |
| (34) | [NCT02926950](https://clinicaltrials.gov/study/NCT02926950) | None identified | Placebo | 2 weeks | Single-blind | Not specified  Not specified  519 randomised |
| (35) | [NCT02743949](https://clinicaltrials.gov/study/NCT02743949) | None identified | Active (first 4 weeks) and placebo (last 2 weeks) | 6 weeks | Single-blind | Not specified  Not specified  256 randomised |
| (36) | [NCT02954848](https://clinicaltrials.gov/study/NCT02954848) | (67) | Placebo | 1 week | Single-blind | 721 enrolled  237 excluded  484 randomised |
| (37) | [NCT02741271](https://clinicaltrials.gov/study/NCT02741271) | (68) | Active | 2 weeks | Open-label | Not specified  Not specified  182 randomised |
| (38) | [NCT02921386](https://clinicaltrials.gov/study/NCT02921386) | None identified | Active | 2 weeks | Not reported | Not specified  Not specified  18 randomised |
| (39) | [NCT02897349](https://clinicaltrials.gov/study/NCT02897349) | None identified | Placebo | 2 weeks | Not reported | Not specified  Not specified  206 randomised |
| (40) | [NCT03242252](https://clinicaltrials.gov/study/NCT03242252) | None identified | Placebo | 2 weeks | Single-blind | Not specified  Not specified  787 randomised |
| (41) | [NCT03351478](https://clinicaltrials.gov/study/NCT03351478) | None identified | Placebo | 2 weeks | Single-blind | Not specified  Not specified  770 randomised |
| (42) | [NCT03285594](https://clinicaltrials.gov/study/NCT03285594) | None identified | Placebo | 4 weeks | Single-blind | Not specified  Not specified  571 randomised |
| (43) | [NCT03242018](https://clinicaltrials.gov/study/NCT03242018) | (69) | Placebo | 2 weeks | Single-blind | Not specified  Not specified  277 randomised |
| (44) | [NCT03066830](https://clinicaltrials.gov/study/NCT03066830) | None identified | Placebo | 2 weeks | Single-blind | Not specified  Not specified  507 randomised |
| (45) | [NCT03332771](https://clinicaltrials.gov/study/NCT03332771) | None identified | Placebo | 2 weeks | Single-blind | 954 enrolled  2 excluded  952 randomised |
| (46) | [NCT03248128](https://clinicaltrials.gov/study/NCT03248128) | None identified | Active | 4 weeks | Open-label | Not specified  Not specified  906 randomised |
| (47) | [NCT03280550](https://clinicaltrials.gov/study/NCT03280550) | (70,71)  ‌ | Active | 5 weeks | Not reported | Not specified  Not specified  138 randomised |
| (48) | [NCT03280537](https://clinicaltrials.gov/study/NCT03280537) | (70,71)  ‌ | Active | 5 weeks | Not reported | Not specified  Not specified  127 randomised |
| (49) | [NCT03023878](https://clinicaltrials.gov/study/NCT03023878) | (72) | Active | Approximately 21 weeks | Not reported. | 47 enrolled,  19 excluded,  28 randomised. |
| (50) | [NCT03429543](https://clinicaltrials.gov/study/NCT03429543) | (73)‌ | Placebo | 2 weeks | Open-label | Not specified  Not specified  158 randomized |
| (51) | [NCT03683576](https://clinicaltrials.gov/study/NCT03683576) | (74) | Active | Minimum of 2 weeks and maximum of 6 weeks | Not reported | 731 enrolled  250 excluded  481 randomised |
| (52) | [NCT03611010](https://clinicaltrials.gov/study/NCT03611010) | None identified | Active | 5 weeks | Not reported | 40 enrolled  0 excluded  40 enrolled into main study (not randomised) |
| (53) | [NCT03614923](https://clinicaltrials.gov/study/NCT03614923) | None identified | Active | Minimum of 20 days and maximum of 31 days | Not reported | Not specified  Not specified  105 randomised |
| (54) | [NCT03792750](https://clinicaltrials.gov/study/NCT03792750) | None identified | Active | 2 weeks | Not reported | Not specified  Not specified  12 enrolled into the main study (no randomised) |
| (55) | [NCT03334253](https://clinicaltrials.gov/study/NCT03334253) | (75) | Placebo | 2-4 weeks | Not reported | 200 enrolled  13 excluded  187 randomised |
| (56) | [NCT04046939](https://clinicaltrials.gov/study/NCT04046939) | (76) | Placebo | Minimum of 12 days and maximum of 30 days | Open-label | 144 enrolled  41 excluded  103 randomised |
| (57) | [NCT04026165](https://clinicaltrials.gov/study/NCT04026165) | None identified | Placebo (for at least 7 days) and active (for at least 4 weeks) | At least 5 weeks | Open-label | 384 (placebo run-in) and  357 (active run-in) enrolled  74 excluded  310 randomised |

SAP; Statistical analysis plan, eDiary; Electronic diary.

References used in supplementary appendix

1. NCT03937479. Verona Pharma plc. A Phase II, Randomized, Double-Blind, Placebo Controlled Dose Ranging Study to Assess the Effect of RPL554 Added on to Tiotropium in Patients With Chronic Obstructive Pulmonary Disease [Internet]. clinicaltrials.gov; 2020 Nov [cited 2024 Oct 22]. Available from: https://clinicaltrials.gov/study/NCT03937479
2. NCT02495168. Actavis Inc. A Randomized, Blinded, Parallel Group, Placebo-Controlled, Multiple Dose, Multicenter, Multinational Study to Compare the Therapeutic Equivalence of a Budesonide 80 μg&#x2F;Formoterol Fumarate Dihydrate 4.5 μg Inhalation Aerosol (Manufactured by Catalent for Watson Laboratories Inc.) to Symbicort® (Budesonide 80 μg&#x2F;Formoterol Fumarate Dihydrate 4.5 μg Inhalation Aerosol) (Manufactured by AstraZeneca) in Adolescent and Adult Patients With Asthma [Internet]. clinicaltrials.gov; 2019 Nov [cited 2024 Oct 22]. Available from: https://clinicaltrials.gov/study/NCT02495168
3. NCT02603809. Idorsia Pharmaceuticals Ltd. A Multi-center, Double-blind, Double-dummy, Randomized, Placebo- and Active-reference, Parallel Group, Phase 2, Dose-finding Study With ACT-132577 in Subjects With Essential Hypertension (Grade 1 and 2). [Internet]. clinicaltrials.gov; 2022 Nov [cited 2024 Oct 22]. Available from: https://clinicaltrials.gov/study/NCT02603809
4. NCT02414958. Boehringer Ingelheim. A Phase III, Randomised, Double Blind, Placebo-controlled, Parallel Group, Efficacy, Safety and Tolerability Trial of Once Daily, Oral Doses of Empagliflozin as Adjunctive to inSulin thErapy Over 52 Weeks in Patients With Type 1 Diabetes Mellitus (EASE-2) [Internet]. clinicaltrials.gov; 2018 Dec [cited 2024 Oct 22]. Available from: https://clinicaltrials.gov/study/NCT02414958
5. NCT02480764. Takeda. A Phase 3, Double-Blind, Randomized, Parallel-Group Study to Compare the Efficacy and Safety of TAK-491 With Valsartan in Chinese Subjects With Essential Hypertension [Internet]. clinicaltrials.gov; 2019 Feb [cited 2024 Oct 22]. Available from: https://clinicaltrials.gov/study/NCT02480764
6. NCT02373813. Amgen. A Randomized Withdrawal Double-blind Study of Etanercept Monotherapy Compared to Methotrexate Monotherapy for Maintenance of Remission in Subjects With Rheumatoid Arthritis [Internet]. clinicaltrials.gov; 2023 Jan [cited 2024 Oct 22]. Available from: https://clinicaltrials.gov/study/NCT02373813
7. NCT02311673. Rhythm Pharmaceuticals, Inc. A Phase 2, Randomized, Double-Blind, Placebo-controlled Pilot Study to Assess the Effects of RM-493, a Melanocortin 4 Receptor (MC4R) Agonist, in Obese Subjects With Prader-Willi Syndrome (PWS) on Safety, Weight Reduction, and Food-Related Behaviors [Internet]. clinicaltrials.gov; 2023 Jul [cited 2024 Oct 22]. Available from: https://clinicaltrials.gov/study/NCT02311673
8. NCT02791438. Takeda. A Phase 3, Open-label, Multicenter, Long-term Study to Evaluate the Safety, Efficacy and Pharmacokinetics of TAK-536 in Pediatric Patients 6 to Less Than 16 Years of Age With Hypertension [Internet]. clinicaltrials.gov; 2020 Feb [cited 2024 Oct 22]. Available from: https://clinicaltrials.gov/study/NCT02791438
9. NCT02926937. Lexicon Pharmaceuticals. A Randomized, Double-blind, Placebo-controlled, Parallel-group, Multicenter Study to Evaluate the Efficacy and Safety of Sotagliflozin as Monotherapy in Patients With Type 2 Diabetes Mellitus Who Have Inadequate Glycemic Control [Internet]. clinicaltrials.gov; 2021 Jun [cited 2024 Oct 22]. Available from: https://clinicaltrials.gov/study/NCT02926937
10. NCT02720081. Merck Sharp &amp; Dohme LLC. A Phase-II, Randomized, Placebo-Controlled, Parallel-Group Clinical Trial to Study the Efficacy and Safety of MK-1029 in Adult Subjects With Persistent Asthma That is Uncontrolled While Receiving Montelukast. [Internet]. clinicaltrials.gov; 2018 Aug [cited 2024 Oct 22]. Available from: https://clinicaltrials.gov/study/NCT02720081
11. NCT02926950. Lexicon Pharmaceuticals. A Randomized, Double-blind, Placebo-controlled, Parallel-group, Multicenter Study to Evaluate the Efficacy and Safety of Sotagliflozin Added to Metformin in Patients With Type 2 Diabetes Mellitus Who Have Inadequate Glycemic Control on Metformin [Internet]. clinicaltrials.gov; 2021 Apr [cited 2024 Oct 22]. Available from: https://clinicaltrials.gov/study/NCT02926950
12. NCT02743949. Takeda. A Randomized, Double-Blind, Proof-of-Concept, Phase 2 Study to Evaluate the Efficacy and Safety of Once Daily Oral Vonoprazan 20 mg or Vonoprazan 40 mg Compared to Esomeprazole 40 mg for the Treatment of Subjects With Symptomatic Gastro-Esophageal Reflux Disease Who Have a Partial Response Following Treatment With a High Dose of Proton Pump Inhibitor [Internet]. clinicaltrials.gov; 2020 Feb [cited 2024 Oct 22]. Available from: https://clinicaltrials.gov/study/NCT02743949
13. NCT02954848. Takeda. A Randomized, Double-Blind, Placebo-Controlled, Multicenter, Phase 3 Study to Evaluate the Efficacy and Safety of Oral TAK-438 10 mg Once-daily in the Treatment of Non-Erosive Gastroesophageal Reflux Disease [Internet]. clinicaltrials.gov; 2019 Jun [cited 2024 Oct 22]. Available from: https://clinicaltrials.gov/study/NCT02954848
14. NCT02741271. Organon and Co. A Phase III, Randomized, Active-Controlled, Parallel-Group Clinical Trial to Study the Efficacy and Long-Term Safety of Mometasone Furoate&#x2F;Formoterol Fumarate (MF&#x2F;F, MK-0887A [SCH418131]), Compared With Mometasone Furoate (MF, MK-0887 [SCH032088]), in Children With Persistent Asthma [Internet]. clinicaltrials.gov; 2024 May [cited 2024 Oct 22]. Available from: https://clinicaltrials.gov/study/NCT02741271
15. NCT02921386. Clarus Therapeutics, Inc. A Phase 2 Study of the Effect of Meals With Various Amounts of Fat Given Immediately After Dosing on the Pharmacokinetics of an Oral Testosterone Undecanoate [Internet]. clinicaltrials.gov; 2018 Apr [cited 2024 Oct 22]. Report No.: NCT02921386. Available from: https://clinicaltrials.gov/study/NCT02921386
16. NCT02897349. Boehringer Ingelheim. A Phase III, Randomised, Double-blind, Placebo-controlled, Parallel Group, Efficacy and Safety Study of Linagliptin, Administered Orally Once Daily, in Combination With Insulin Therapy for 24 Weeks in Chinese Type 2 Diabetes Mellitus Patients With Insufficient Glycaemic Control [Internet]. clinicaltrials.gov; 2020 Mar [cited 2024 Oct 22]. Available from: https://clinicaltrials.gov/study/NCT02897349
17. NCT03242252. Lexicon Pharmaceuticals. A Randomized, Double-blind, Placebo-controlled, 3-arm, Parallel-group 52-week Multicenter Study to Evaluate the Efficacy and Safety of Sotagliflozin in Patients With Type 2 Diabetes Mellitus and Moderate Renal Impairment Who Have Inadequate Glycemic Control [Internet]. clinicaltrials.gov; 2021 Jun [cited 2024 Oct 22]. Available from: https://clinicaltrials.gov/study/NCT03242252
18. NCT03351478. Lexicon Pharmaceuticals. A 26-week Randomized, Double-blind, Controlled, Parallel-group, Multicenter Study to Evaluate the Efficacy and Safety of Sotagliflozin Compared to Empagliflozin, and Placebo in Participants With Type 2 Diabetes Who Have Inadequate Glycemic Control on Dipeptidyl Peptidase 4 Inhibitor (DPP4(i)) With or Without Metformin [Internet]. clinicaltrials.gov; 2021 Apr [cited 2024 Oct 22]. Available from: https://clinicaltrials.gov/study/NCT03351478
19. NCT03285594. Lexicon Pharmaceuticals. A Randomized, Double-blind, Placebo-controlled, Parallel-group, 52-week Multicenter Study to Evaluate the Efficacy and Safety of Sotagliflozin in Patients With Type 2 Diabetes Who Have Inadequate Glycemic Control on Basal Insulin Alone or in Addition to Oral Antidiabetes Drugs (OADs) [Internet]. clinicaltrials.gov; 2021 Apr [cited 2024 Oct 22]. Available from: https://clinicaltrials.gov/study/NCT03285594
20. NCT03242018. Lexicon Pharmaceuticals. A Randomized, Double-blind, Placebo-controlled, 3-arm, Parallel-group 52-week Multicenter Study to Evaluate the Efficacy and Safety of Sotagliflozin in Patients With Type 2 Diabetes Mellitus and Severe Renal Impairment Who Have Inadequate Glycemic Control [Internet]. clinicaltrials.gov; 2021 Jun [cited 2024 Oct 22]. Available from: https://clinicaltrials.gov/study/NCT03242018
21. NCT03066830. Lexicon Pharmaceuticals. A Randomized, Double-blind, Placebo-controlled, Parallel-group, Multicenter Study to Evaluate the Efficacy and Safety of Sotagliflozin Added to a Sulfonylurea Alone or in Combination With Metformin in Patients With Type 2 Diabetes Who Have Inadequate Glycemic Control on a Sulfonylurea Alone or With Metformin [Internet]. clinicaltrials.gov; 2021 Apr [cited 2024 Oct 22]. Available from: https://clinicaltrials.gov/study/NCT03066830
22. NCT03332771. Lexicon Pharmaceuticals. A 52-week Randomized, Double-blind, Double-dummy, Active and Placebo-controlled, Parallel-group, Multicenter Study to Evaluate the Efficacy and Safety of Sotagliflozin Compared to Glimepiride or Placebo Added to Metformin in Patients With Type 2 Diabetes Who Have Inadequate Glycemic Control With Metformin Monotherapy [Internet]. clinicaltrials.gov; 2021 Apr [cited 2024 Oct 22]. Available from: https://clinicaltrials.gov/study/NCT03332771
23. NCT03248128. GlaxoSmithKline. A Randomised, Double-blind, Parallel Group, Multicentre, Stratified, Study Evaluating the Efficacy and Safety of Once Daily Fluticasone Furoate&#x2F;Vilanterol Inhalation Powder Compared to Once Daily Fluticasone Furoate Inhalation Powder in the Treatment of Asthma in Participants Aged 5 to 17 Years Old (Inclusive) Currently Uncontrolled on Inhaled Corticosteroids [Internet]. clinicaltrials.gov; 2023 May [cited 2024 Oct 22]. Available from: https://clinicaltrials.gov/study/NCT03248128
24. NCT03280550. Hoffmann-La Roche. A Phase III, Randomized, Multicenter, Double-blind, Placebo-controlled Clinical Trial of Omalizumab in Patients With Chronic Rhinosinusitis With Nasal Polyps [Internet]. clinicaltrials.gov; 2020 Mar [cited 2024 Oct 22]. Available from: https://clinicaltrials.gov/study/NCT03280550
25. NCT03280537. Hoffmann-La Roche. A Phase III, Randomized, Multicenter, Double-blind, Placebo-controlled Clinical Trial of Omalizumab in Patients With Chronic Rhinosinusitis With Nasal Polyps [Internet]. clinicaltrials.gov; 2020 Mar [cited 2024 Oct 22]. Available from: https://clinicaltrials.gov/study/NCT03280537
26. NCT03023878. Amgen. 20150288 A Phase 2 Open-label Study Investigating the Safety and Efficacy of Blinatumomab After Frontline R-Chemotherapy in Adult Subjects With Newly Diagnosed High-risk Diffuse Large B-Cell Lymphoma (DLBCL) [Internet]. clinicaltrials.gov; 2020 Aug [cited 2024 Oct 22]. Available from: https://clinicaltrials.gov/study/NCT03023878
27. NCT03429543. Boehringer Ingelheim. A Double-blind, Randomised, Placebo-controlled, Parallel Group Trial to Evaluate the Efficacy and Safety of Empagliflozin and Linagliptin Over 26 Weeks, With a Double-blind Active Treatment Safety Extension Period up to 52 Weeks, in Children and Adolescents With Type 2 Diabetes Mellitus [Internet]. clinicaltrials.gov; 2024 Feb [cited 2024 Oct 22]. Available from: https://clinicaltrials.gov/study/NCT03429543
28. NCT03683576. GB001, Inc, a wholly owned subsidiary of Gossamer Bio, Inc. A Phase 2b, Randomized, Double-blind, Placebo-controlled, Dose-ranging, Multi-center Study to Evaluate the Efficacy and Safety of GB001 as Maintenance Therapy in Adult Subjects With Moderate to Severe Asthma [Internet]. clinicaltrials.gov; 2021 Aug [cited 2024 Oct 22]. Available from: https://clinicaltrials.gov/study/NCT03683576
29. NCT03611010. Cumberland Pharmaceuticals. Phase II, Dose-ranging Study to Evaluate the Efficacy Dose Response and Pharmacokinetics of Intravenous Atorvastatin in Hypercholesterolemic Patients Previously Controlled With Oral Atorvastatin [Internet]. clinicaltrials.gov; 2022 Jun [cited 2024 Oct 22]. Available from: https://clinicaltrials.gov/study/NCT03611010
30. NCT03614923. AnaptysBio, Inc. A Phase 2, Double-Blind, Placebo-Controlled, Parallel Group, Multiple Dose Study to Investigate Etokimab (ANB020) in Adult Subjects With Chronic Rhinosinusitis With Nasal Polyposis [Internet]. clinicaltrials.gov; 2022 Jan [cited 2024 Oct 22]. Available from: https://clinicaltrials.gov/study/NCT03614923
31. NCT03792750. Bristol-Myers Squibb. A Phase 1&#x2F;2 Study to Evaluate the Pharmacokinetics, Pharmacodynamics, Safety, and Tolerability of BMS-986205 Alone and in Combination With Nivolumab in Chinese Patients With Advanced Malignant Solid Tumors [Internet]. clinicaltrials.gov; 2022 Feb [cited 2024 Oct 22]. Available from: https://clinicaltrials.gov/study/NCT03792750
32. NCT03334253. Jaeb Center for Health Research. Low-Dose Atropine for Treatment of Myopia (Myopia Treatment Study) [Internet]. clinicaltrials.gov; 2023 Aug [cited 2024 Oct 22]. Available from: https://clinicaltrials.gov/study/NCT03334253
33. NCT04046939. Knopp Biosciences. A Randomized, Double-Blind, Placebo-Controlled Dose-Ranging Biomarker Study of the Effects of Dexpramipexole on Eosinophils in Subjects With Eosinophilic Asthma [Internet]. clinicaltrials.gov; 2023 Apr [cited 2024 Oct 22]. Available from: https://clinicaltrials.gov/study/NCT04046939
34. NCT04026165. Gilead Sciences. MOSAIC - A Phase 2b, Randomized, Double-Blind, Placebo-Controlled, Parallel Group, Multicenter Study Evaluating the Efficacy and Safety of Selonsertib in Subjects With Moderate to Advanced Diabetic Kidney Disease [Internet]. clinicaltrials.gov; 2022 Nov [cited 2024 Oct 22]. Available from: https://clinicaltrials.gov/study/NCT04026165
35. Ferguson GT, Kerwin EM, Rheault T, Bengtsson T, Rickard K. A Dose-Ranging Study of the Novel Inhaled Dual PDE 3 and 4 Inhibitor Ensifentrine in Patients with COPD Receiving Maintenance Tiotropium Therapy. COPD. 2021 Apr;Volume 16:1137–48.
36. Verweij P, Danaietash P, Flamion B, Ménard J, Bellet M. Randomized Dose-Response Study of the New Dual Endothelin Receptor Antagonist Aprocitentan in Hypertension. Hypertension. 2020 Apr;75(4):956–65.
37. Rosenstock J, Marquard J, Laffel LM, Neubacher D, Kaspers S, Cherney DZ, et al. Empagliflozin as Adjunctive to Insulin Therapy in Type 1 Diabetes: The EASE Trials. Diabetes Care. 2018 Dec 1;41(12):2560–9.
38. Song C, Dhaliwal S, Bapat P, Scarr D, Bakhsh A, Budhram D, et al. Point-of-Care Capillary Blood Ketone Measurements and the Prediction of Future Ketoacidosis Risk in Type 1 Diabetes. Diabetes Care. 2023 Nov 1;46(11):1973–7.
39. Wu J, Du X, Lv Q, Li Z, Zheng Z, Xia Y, et al. A phase 3 double-blind randomized (CONSORT-compliant) study of azilsartan medoxomil compared to valsartan in Chinese patients with essential hypertension. Medicine (Baltimore). 2020 Aug 7;99(32):e21465.
40. Curtis JR, Stolshek B, Emery P, Haraoui B, Karis E, Kricorian G, et al. Effects of Disease-Worsening Following Withdrawal of Etanercept or Methotrexate on Patient-Reported Outcomes in Patients With Rheumatoid Arthritis: Results From the SEAM-RA Trial. J Clin Rheumatol. 2023 Jan 1;29(1):16–22.
41. Curtis JR, Emery P, Karis E, Haraoui B, Bykerk V, Yen PK, et al. Etanercept or Methotrexate Withdrawal in Rheumatoid Arthritis Patients in Sustained Remission. Arthritis & Rheumatology. 2021 May;73(5):759–68.
42. Curtis JR, Trivedi M, Haraoui B, Emery P, Park GS, Collier DH, et al. Defining and characterizing sustained remission in patients with rheumatoid arthritis. Clin Rheumatol. 2018 Apr;37(4):885–93.
43. Curtis JR, Emery P, Kricorian G, Yen PK, Collier DH, Bykerk V, et al. Factors Associated With Maintenance of Remission Following Change From Combination Therapy to Monotherapy in Patients With Rheumatoid Arthritis. J Rheumatol. 2023 Sep;50(9):1114–20.
44. Kinoshita Y, Sakurai Y, Takabayashi N, Kudou K, Araki T, Miyagi T, et al. Efficacy and Safety of Vonoprazan in Patients With Nonerosive Gastroesophageal Reflux Disease: A Randomized, Placebo-Controlled, Phase 3 Study. Clin Transl Gastroenterol. 2019 Nov;10(11):e00101.
45. Weinstein CLJ, Gates D, Zhang X, Varnell T, Mok W, Vermeulen JH, et al. A phase 3 study evaluating the safety and efficacy of a pediatric dose of mometasone furoate with and without formoterol for persistent asthma. Pediatric Pulmonology. 2020 Apr;55(4):882–9.
46. Cherney DZI, Ferrannini E, Umpierrez GE, Peters AL, Rosenstock J, Carroll AK, et al. Efficacy and safety of sotagliflozin in patients with type 2 diabetes and severe renal impairment. Diabetes Obesity Metabolism. 2021 Dec;23(12):2632–42.
47. Gevaert P, Omachi TA, Corren J, Mullol J, Han J, Lee SE, et al. Efficacy and safety of omalizumab in nasal polyposis: 2 randomized phase 3 trials. Journal of Allergy and Clinical Immunology. 2020 Sep;146(3):595–605.
48. Damask C, Chen M, Holweg CTJ, Yoo B, Millette LA, Franzese C. Defining the Efficacy of Omalizumab in Nasal Polyposis: A POLYP 1 and POLYP 2 Subgroup Analysis. Am J Rhinol Allergy. 2022 Jan;36(1):135–41.
49. Katz DA, Morris JD, Chu MP, David KA, Thieblemont C, Morley NJ, et al. Open-label, phase 2 study of blinatumomab after frontline R-chemotherapy in adults with newly diagnosed, high-risk DLBCL. Leukemia & Lymphoma. 2022 Jul 29;63(9):2063–73.
50. Laffel LM, Danne T, Klingensmith GJ, Tamborlane WV, Willi S, Zeitler P, et al. Efficacy and safety of the SGLT2 inhibitor empagliflozin versus placebo and the DPP-4 inhibitor linagliptin versus placebo in young people with type 2 diabetes (DINAMO): a multicentre, randomised, double-blind, parallel group, phase 3 trial. The Lancet Diabetes & Endocrinology. 2023 Mar;11(3):169–81.
51. Moss MH, Lugogo NL, Castro M, Hanania NA, Ludwig-Sengpiel A, Saralaya D, et al. Results of a Phase 2b Trial With GB001, a Prostaglandin D2 Receptor 2 Antagonist, in Moderate to Severe Eosinophilic Asthma. CHEST. 2022 Aug;162(2):297–308.
52. Repka MX, Weise KK, Chandler DL, Wu R, Melia BM, Manny RE, et al. Low-Dose 0.01% Atropine Eye Drops vs Placebo for Myopia Control: A Randomized Clinical Trial. JAMA Ophthalmol. 2023 Aug 1;141(8):756.
53. Siddiqui S, Wenzel SE, Bozik ME, Archibald DG, Dworetzky SI, Mather JL, et al. Safety and Efficacy of Dexpramipexole in Eosinophilic Asthma (EXHALE): A randomized controlled trial. Journal of Allergy and Clinical Immunology. 2023 Nov;152(5):1121-1130.e10.
